# Orbital Metastasis of Breast Cancer Mimicking Invasive Fungal Rhinosinusitis

**DOI:** 10.1155/2016/2913241

**Published:** 2016-08-01

**Authors:** Mayara Tabai, Igor Moreira Hazboun, Emerson Taro Inoue Sakuma, Marcelo Hamilton Sampaio, Eulalia Sakano

**Affiliations:** Department of Otolaryngology Head and Neck, Faculty of Medical Sciences, University of Campinas (UNICAMP), P.O. Box 6111, 13081-970 Campinas, SP, Brazil

## Abstract

*Introduction*. A range of traumatic, vascular, inflammatory, infectious, and neoplastic processes can affect the orbit and its structures. In the area of otolaryngology, the rhino-orbital-cerebral involvement of invasive fungal rhinosinusitis can affect the orbit, which may look like initially a rhinosinusitis or even mimic malignancy.* Case Presentation*. Female patient, 32 years old, with headache and ocular proptosis. She was using prednisone in immunosuppressive doses for a year and had breast cancer treated three years earlier. The initial CT scan showed opacification of the sphenoid and ethmoid sinuses, left intraorbital involvement and contrast impregnation in the cavernous sinus. The biopsy resulted positive for invasive ductal carcinoma of the breast.* Discussion*. The initial CT scan of our patient showed both signs of early changes of invasive fungal rhinosinusitis (IFR) and possible metastatic involvement. The intracranial extension and ocular involvement are usually the most common signs of IFR (first hypothesis). Among metastases at the orbit and the eye, breast and lung carcinomas are the most frequent.* Conclusion*. Although several studies on the differential diagnosis of orbital lesions exist, especially when it concerns the involvement of the nasal cavity, the diagnosis by imaging is still a challenge.

## 1. Introduction

Paranasal sinuses and orbit are potential sites for metastases. Distant metastases of skull base occur in 4% of patients with cancer and the most common primary sites are breast, prostate, and lung. Occasionally, breast carcinoma can metastasize to the nasal cavity and orbit and manifest with unspecific symptoms. If the primary tumor is unknown and the metastatic deposits mimic rhinosinusitis or cause cavernous sinus syndrome, the diagnosis is usually delayed. Although metastasis to the orbit is rare, it must be considered in the differential diagnosis of any patient with history of cancer, presenting with ophthalmic symptom [1].

Orbital metastasis constitutes 3% of orbital lesions and 10% of orbital tumors. Breast cancer is the most common primary site for orbital metastasis in women with known disseminated disease and the ocular symptoms can appear years after the inicial brest cancer diagnosis [2]. Considering all types of orbital metastasis, in 19%, there is no history of cancer when the patient presents with ophthalmic symptoms and, in 10%, the primary site remains unclear, despite systemic evaluation. Computed tomography or magnetic resonance imaging can show only a thickening of the extraocular muscles, and often the possibility of an inflammatory process is raised [3].

A careful history and physical examination, with special attention to the orbit and eye attachments, are necessary to identify subtle orbital anomalies that might otherwise be ignored or mistakenly contributed to a nonorbital process. In this context, we can mention, in the area of otolaryngology, the rhino-orbital-cerebral involvement of invasive fungal rhinosinusitis, especially in immunocompromised patients [4].

Clinically, rhino-orbital involvement initially may look like a rhinosinusitis or even mimic malignancy. Also, we must consider the differential diagnosis of orbital disorders, such as migraine; chronic rhinosinusitis; preseptal and orbital cellulitis; primary and secondary orbital tumor (metastasis); posttraumatic hematoma; inflammatory pseudotumor; thrombosis of the cavernous sinus; and Graves' disease.

This paper aims to report a case of orbital metastasis of breast cancer mimicking invasive fungal rhinosinusitis.

## 2. Case Report

Female patient, 32 years old, has been complaining about a headache for a month, with worsening pain and ocular proptosis a week before, associated with hyaline nasal discharge. A throbbing headache with progression was located in the left frontal region, associated with fatigue and myalgia. She also reported proptosis on the left eye, followed by diplopia and eye pain.

Regarding her medical history, the patient had triple negative breast adenocarcinoma treated with radical mastectomy and chemotherapy three years earlier. Moreover, the patient had atopic dermatitis, which was difficult to control, diagnosed a year before, using prednisone in immunosuppressive doses (80 mg/day in the last year), also with controlled asthma and hypertension.

The physical examination on the patient showed regular condition, normal vital signs, facies cushingoid, and left ocular proptosis. Nasal endoscopy only indicated a discrete hyaline secretion in the nasal vestibule.

**Figure 1:**
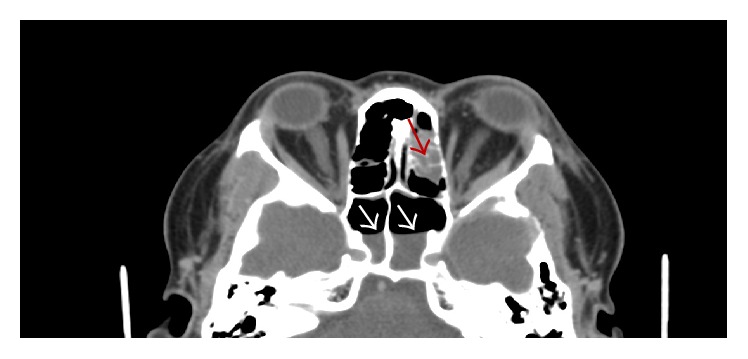
CT with contrast in the sinuses demonstrates opacification of certain ethmoid cells (red arrow) and fluid level in the sphenoid sinus bilaterally (white arrow).

**Figure 2:**
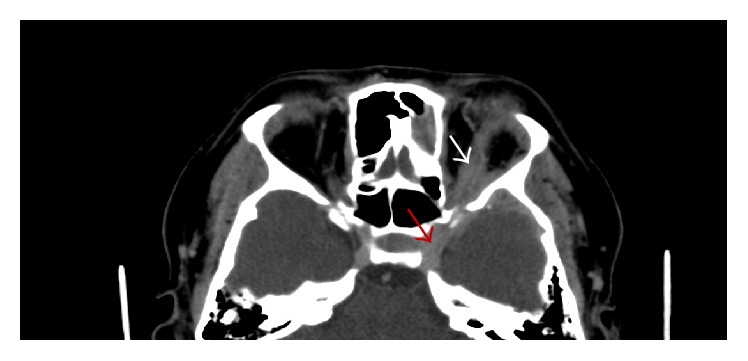
CT with contrast in the sinuses demonstrates left intraorbital involvement (white arrow) that extends through the cavernous sinus determining pachymeningeal enhancement (red arrow).

Faced with this situation, we opted to perform Computerized Tomography of the skull and sinuses. The initial CT scan revealed discreet parenchymal atrophy, opacification of the sphenoid and ethmoid sinuses bilaterally, and left intraorbital involvement with contrast impregnation that extends to the intracranial region through the cavernous sinus determining pachymeningeal enhancement (Figures 1 and 2).

After discussion, we got the hypothesis that it was invasive fungal rhinosinusitis with involvement of the cavernous sinus; then we scheduled a biopsy and gave intravenous Amphotericin B. One day later, the patient developed worsening of proptosis and ocular motility dysfunction and reached oculomotor nerve palsy.

**Figure 3:**
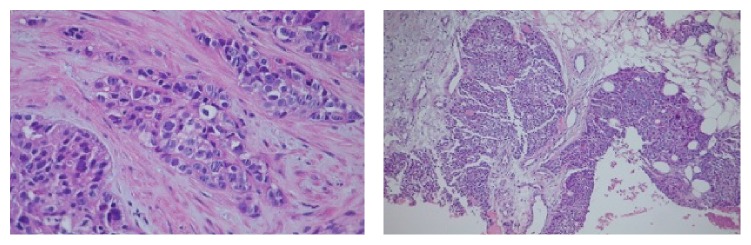
Microscopic pathology image showing malignant cells resulting in metastatic carcinoma of breast. H & E stain.

Then the case was discussed with staff of Radiology, who guided the achievement of chest CT. In the new images were visualized a nodule in the right breast of 1.6 cm with regular borders, mediastinal lymph nodes up to 2.2 cm, peribronchial mass (lymph node conglomerate), and atelectasis in the left lung. The orbital and lymph nodes biopsy tested positive for invasive ductal carcinoma of the breast (Figure 3). The patient was referred for radiotherapy and chemotherapy. After initiating chemotherapy, the patient presented clinical instability and died, not being possible to perform additional imaging studies.

## 3. Discussion

Breast cancer may metastasize to uncommon anatomic sites, but there are no solid statistical data concerning the frequency of head and neck metastasis in cancer patients. Some authors suggest that histologically confirmed orbital metastasis is diagnosed once or twice per year at larger clinical centers. These patients often complain of ocular asymmetry (noticed by the patient) or diplopia, without visual field impairment. Depending on the series, breast cancers account for 29–53% of orbital metastasis and the infiltrating lobular breast cancer is the most common subtype (87.5%) [5]. The fact that our patients present a ductal breast cancer makes our case even rarer. The presence of ocular metastasis is a bad prognostic indicator, with survival that can range from 0 to 64 months, with an average of five months [6].

The extraocular muscles represent the main site of breast cancer orbital metastasis, causing pain, proptosis, and diplopia. They are identified pathologically as solid deposits of the muscles. Orbital metastasis may cause exophthalmos, from mass effect, or enophthalmos [7]. Metastatic breast cancer rarely can manifest as cranial nerve palsies (incidence of 0.13%). The most frequently affected cranial nerves are V (70%) and VII (60%). Rarely, cavernous sinus metastasis can be the first presentation of an undetected breast cancer. The cavernous sinus can be affected by inflammatory, vascular, or neoplastic conditions. Neoplasm may arise from adjacent structures or from distant metastasis [8].

There have been four previous case reports of cavernous sinus syndrome as the first presentation of metastatic breast cancer. However, the initial clinical presentation of these cases varies: Ryan et al. reported a patient who presented with headache and painful proptosis [9]; Martín Polo et al. reported a patient with pain and numbness over the left side of face with occasional diplopia [10]; Fyrmpas et al. published a case who presented with rhino-orbital cellulites [1]; and Khaw et al. reported a patient with ptosis due to cavernous sinus syndrome as a rare presentation of advanced breast metastasis [11].

Rhino-orbital-cerebral infection is the most common clinical presentation of mucormycosis. This infection usually presents as acute sinusitis with fever, nasal congestion, purulent nasal discharge, headache, and sinus pain. However, there have been certain reports of rhino-orbital-cerebral mucormycosis with an indolent course. The most common presenting features of an indolent invasive fungal rhinosinusitis (IFS) are ophthalmologic and include ptosis, proptosis, visual loss, and ophthalmoplegia. The incidence of internal carotid artery and cavernous sinus involvement is higher in indolent rhino-orbital-cerebral infection than in the acute disease [12]. In our case, we are before a patient with chronic use of corticosteroids in immunosuppressive doses for a year, being considered a risk patient to develop invasive fungal disease [5, 13].

With these clinical manifestations presented by the patient (headache, proptosis, or diplopia), we have to investigate the differential diagnosis of orbital disorders, because differentiating neoplasm injury of the paranasal sinuses or orbit of an IFR may not be possible only through the tomographic imaging, due to similarities that these lesions may present [5, 13, 14].

Usually the initial examinations of patients with acute invasive fungal rhinosinusitis revealed nonspecific mucosal thickening and opacification of the nasal cavity, which cannot be distinguished from a common rhinosinusitis. In a patient with sinusitis, the obliteration or infiltration of the normal fat density within the periantral regions suggests deep tissue extension and often is the earliest sign of IFR [14].

Among metastasis at the orbit and the eye, breast and lung carcinomas are the most frequent. Isolated orbit lesions are rare and more infiltrative and tend to involve multiple sites (choroid, optic nerve, muscle, and extraconal region). In the CT it is possible to see a hyperdense area in comparison to fat and muscles. After administration of intravenous contrast, it demonstrates important heterogeneous enhancement [12].

The initial CT scan of our patient showed both signs of early changes of IFR and possible metastatic involvement, such as opacification of the sphenoid and ethmoid sinuses and intraorbital involvement with contrast impregnation that extends to the intracranial region through the cavernous sinus, which determines pachymeningeal enhancement. The intracranial extension and ocular involvement are usually the most common signs of fungal rhinosinusitis (first hypothesis).

Pachymeningeal (thick meninges) enhancement can manifest against the bone or it may involve the dural reflections of the falx cerebri, tentorium cerebelli, falx cerebelli, and cavernous sinus. Extra-axial pachymeningeal enhancement can arise from various benign or malignant processes, including transient postoperative changes, intracranial hypotension and neoplasms, such as meningiomas, metastatic disease (from breast and prostate cancer), secondary lymphoma, and granulomatous disease. Granulomatous disease (including sarcoid, tuberculosis, Wegener granulomatosis, luetic gummas, and rheumatoid nodules) and fungal disease can produce pachymeningeal enhancement [15].

The veracity of the diagnosis between the two situations must be done by biopsy. IFR is characterized by vascular invasion, hyphae in the submucosa, or tissue necrosis without inflammatory cells. The most common type of breast carcinoma is formed by proliferation of the epithelial elements with cytologic atypia. It has a tendency to form pseudoglandular structures and variable mitotic activity. If the primary tumor is acknowledged, treatment can be instituted, based on the clinical and radiological findings, regardless of histological confirmation [16].

Local treatment's aim is to preserve patients' vision and improve their quality of life. It is administered in addition to systemic treatment regimens. Local treatment modalities include radiotherapy, laser application, intravitreal antivascular endothelial growth factors (anti-VEGF), photodynamic therapy, and enucleation of the eye. Despite recent advances in diagnosis and treatment modalities, the prognosis of breast cancer metastatic disease remains scarce with estimated median life expectancy of 6 to 9 months [7].

## 4. Conclusion

In the invasive fungal rhinosinusitis or in the eye metastasis, the diagnosis should be done quickly due to high morbidity and mortality of these two entities. And although several studies on the differential diagnosis of orbital lesions exist, especially when it concerns the involvement of the nasal cavity, the diagnosis by imaging is still a challenge.
